# The effects of mechanical noise bandwidth on balance across flat and compliant surfaces

**DOI:** 10.1038/s41598-021-91422-w

**Published:** 2021-06-10

**Authors:** Jeshaiah Zhen Syuen Khor, Alpha Agape Gopalai, Boon Leong Lan, Darwin Gouwanda, Siti Anom Ahmad

**Affiliations:** 1 School of Engineering and the Advanced Engineering Platform, Monash University, Selangor, Malaysia; 2 Malaysian Research Institute on Ageing, Universiti Putra Malaysia, Selangor, Malaysia

**Keywords:** Biomedical engineering, Quality of life

## Abstract

Although the application of sub-sensory mechanical noise to the soles of the feet has been shown to enhance balance, there has been no study on how the bandwidth of the noise affects balance. Here, we report a single-blind randomized controlled study on the effects of a narrow and wide bandwidth mechanical noise on healthy young subjects’ sway during quiet standing on firm and compliant surfaces. For the firm surface, there was no improvement in balance for both bandwidths—this may be because the young subjects could already balance near-optimally or optimally on the surface by themselves. For the compliant surface, balance improved with the introduction of wide but not narrow bandwidth noise, and balance is improved for wide compared to narrow bandwidth noise. This could be explained using a simple model, which suggests that adding noise to a sub-threshold pressure stimulus results in markedly different frequency of nerve impulse transmitted to the brain for the narrow and wide bandwidth noise—the frequency is negligible for the former but significantly higher for the latter. Our results suggest that if a person’s standing balance is not optimal (for example, due to aging), it could be improved by applying a wide bandwidth noise to the feet.

## Introduction

Balance is essential to human life because it controls an individual’s ability to carry out activities of daily living. Having a good balance ensures a good quality of life^1^. As an individual’s ability to balance begins to deteriorate the individual becomes increasingly susceptible to falls and trips which may lead to injuries or even death^2–6^. This is especially true for older adults who are aged above 65, where falls have been reported to be a major cause of death^7^. This has led to increased research interest in fall risk assessment^8–10^ and fall detection^11,12^.

The somatosensory system contributes significantly to balance, especially via the mechanoreceptors in the sole of the foot that provide information on changes in pressure^13^ for the body to adjust its posture to maintain balance in real time. Advancing age, disease, and injuries have adverse effects on the sensitivity of the somatosensory system, impacting the ability to balance and hence increasing fall risk^14^. As a result, the potential of enhancing sensitivity in the somatosensory system to improve balance through the application of mechanical noise (to the soles of the feet) has been investigated. The introduction of mechanical noise to the feet reduced sway parameters during quiet standing in healthy young subjects and healthy elderly subjects^15–18^. Similar findings were reported among subjects affected by neurodegenerative conditions such as diabetic neuropathy and stroke-induced hemiparesis^19,20^. The benefits of this treatment were not limited to static postural sway, as positive effects, such as reductions in gait variability, were also observed when noise was applied during ambulation^21,22^. However, some studies have reported no observable positive effects in a portion of their subject group^18,20,23^.

The methods used to produce the mechanical noise varied across previous studies. Firstly, a variety of mechanical vibratory transducers have been used to produce the mechanical noise, ranging from nylon indenters^15,17^ to electromagnetic actuators^17–19^ to piezoelectric elements^20,21,24^. Secondly, different electrical signals have been used to drive the mechanical vibratory transducer. Although white noise is generally used as the driving signal, there are mainly two bandwidths reported in the literature: (1) 1–100 Hz^15–17,19,23,25,26^ and (2) 1–500 Hz^18,20^, while some studies did not specify the bandwidth of the driving signal used^27–29^. These variations in producing mechanical noise is a possible cause as to why there have been conflicting results reported. For example, in Priplata et al.^15^ and Priplata et al.^16^, although the bandwidth of the electrical white noise is the same (1–100 Hz), different mechanical transducers were used. The results from the two studies were inconsistent—some sway parameters increased in Priplata et al.^16^ but not in Priplata et al.^15^ for similar groups of healthy young adults. In another example, the results of Priplata et al.^16^ and Dettmer et al.^18^, which utilized similar mechanical transducers but different electrical white noise bandwidths, were also inconsistent—sway parameters improved for healthy young subjects in Priplata et al.^16^ (1–100 Hz) but not in Dettmer et al.^18^ (1–500 Hz).

The characteristics of the mechanical noise produced by the mechanical transducer depend on both the transducer and the bandwidth of the electrical white noise applied to the transducer. The mechanical noise produced is different for different bandwidths (although with the same transducer) and across different transducers (despite having the same bandwidth). We believe the inconsistent results highlighted above are due to differences in the characteristics of the mechanical noise applied to the feet. Therefore, in this paper, we study the effect of sub-sensory mechanical noise with two different bandwidths: (1) a narrow bandwidth with a low maximum frequency (referred to henceforth as narrow) and (2) a wider bandwidth with a higher maximum frequency (referred to henceforth as wide) on the balance of healthy young subjects during quiet standing on firm and compliant surfaces. The aim of the study was to investigate whether the different bandwidths have different effects on balance.

## Materials and methods

### Subjects

Subjects were recruited from the student population of Monash University Malaysia. The exclusion criteria were: (1) they had a current or history of serious injury, disability, or disease affecting postural control; (2) they were unable to feel introduced supra-threshold vibration at maximal levels for any vibration type used in the study; (3) they were unable to stand unsupported on the compliant surface for longer than two minutes; (4) they had any other conditions rendering them unsuitable for the study in the judgement of the investigators.

All subjects provided informed consent before the commencement of the evaluation in accordance with the Declaration of Helsinki. The subject group for the study consisted of 10 individuals aged 18–24 years; 4 male and 6 female, with a mean age, height, and weight of 21.5 ± 1.4 years, 164.3 ± 7.3 cm, and 56.4 ± 10 kg respectively. The study was reviewed and approved by the Monash University Human Research Ethics Committee.

**Figure 1:**
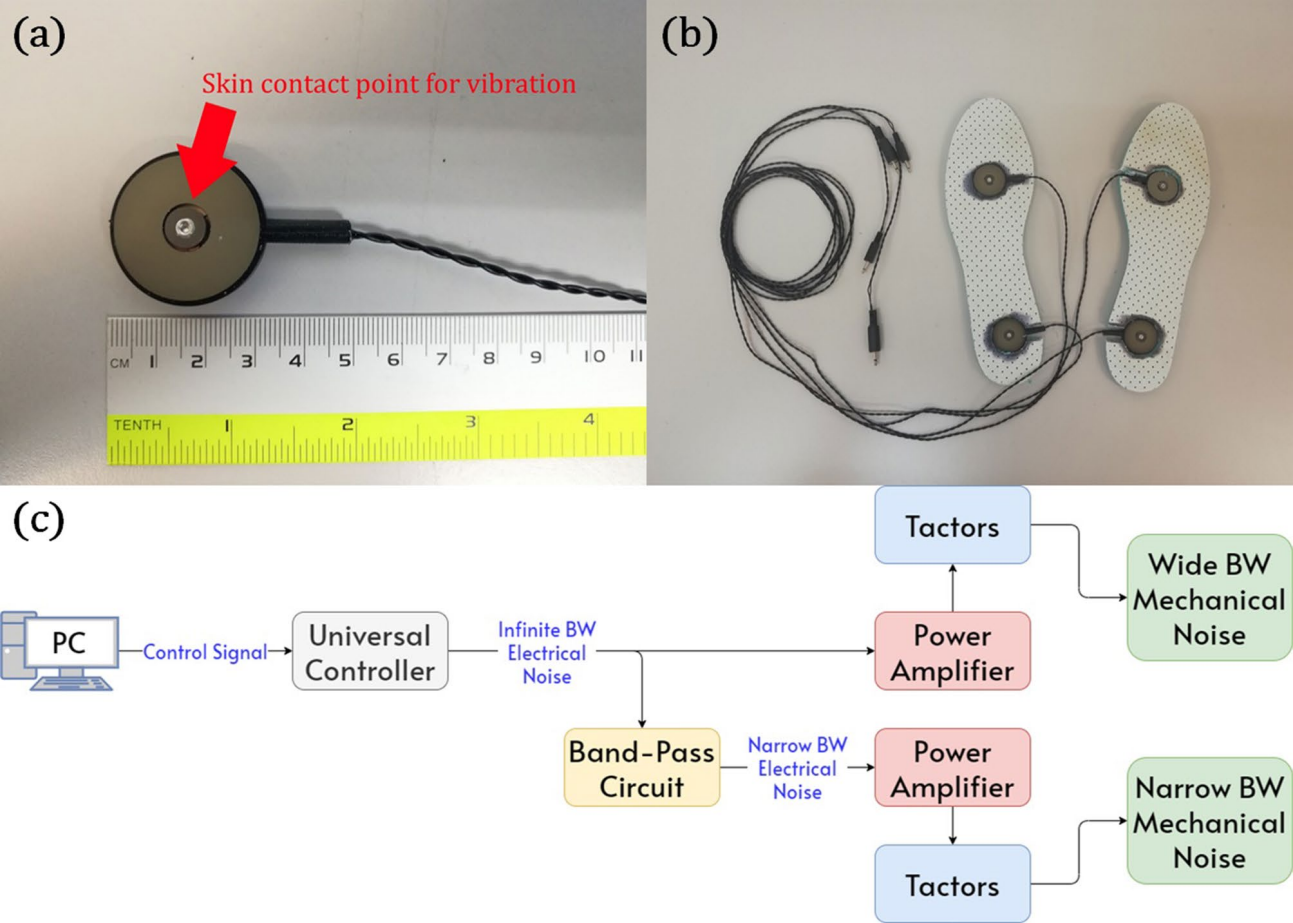
(**a**) Vibrotactile transducers used to generate mechanical noise. (**b**) Top view of insoles with transducers inserted used in balance trials. The vibrating surface of the transducers was flush with the top surface of the insoles. (**c**) Schematic of system used to generate different bandwidths of mechanical noise.

### Mechanical noise generation and delivery system

Sub-sensory mechanical noise was delivered to the plantar side of the feet of each subject via two vibrotactile transducers (C2 tactors; Engineering Acoustics Inc., FL, USA) shown in Fig. 1a, which were embedded in an 8-mm thick fabric insole. The transducers were positioned to be in contact with the forefoot and heel^16,21,26^, as depicted in Fig. 1b. Multiple insole sizes were prepared to cater to varying foot sizes. All insoles used in the study were sanitized before and after each use.

The two bandwidths of mechanical noise used (narrow and wide) were generated by the vibrotactile transducers. The transducers were supplied with electrical white noise from a control box (Universal Controller; Engineering Acoustics Inc., FL, USA) designed for transducer control. Wide bandwidth mechanical noise was produced by the transducers supplied with an infinite bandwidth electrical white noise generated by the control box. The infinite bandwidth electrical noise was chosen as the driving signal as it contained the widest range of frequencies and was the best choice to produce a wide bandwidth of mechanical noise. This wide bandwidth mechanical noise could also be replicated with a finite bandwidth electrical noise with a bandwidth significantly larger than the maximum frequency of the mechanical noise produced by the infinite bandwidth electrical noise. Narrow bandwidth mechanical noise was produced by the transducers when they were supplied with 1–100 Hz band-limited electrical white noise. To obtain this band limited signal, the infinite bandwidth electrical white noise signal from the control box was passed through a band-pass filter (of approximately 1–100 Hz). The band-pass filter was devised using a second order passive high-pass filter and a sixth order active low-pass filter to ensure a high roll-off rate for an accurate bandwidth. Vibration magnitudes for each noise type were adjusted using a PC connected to the universal controller. The schematic of the system used to generate the wide and narrow bandwidth mechanical noises is shown in Fig. 1c.

### Mechanical noise spectrum measurement

To quantify the characteristics of the mechanical noise generated, the vibration profile of the transducers was measured using a tri-axial digital accelerometer (ADXL345, Analog Devices Inc., MA, USA)^30,31^ with a maximum sampling rate of 3200 Hz. The accelerometer was attached to the vibratory site of the transducer using putty, to ensure no additional noise/vibration was introduced to the accelerometer as a result of a poor attachment. A 4-wire serial peripheral interface was used to connect the accelerometer to a Raspberry Pi 3B+ (Raspberry Pi Foundation, CAM, UK) for data transfer.

The frequency spectrum of the measured acceleration was then used to quantify the frequency spectrum of the mechanical noise produced. The power spectral density of the measured raw acceleration values was calculated in MATLAB (ver. R2020a, The Mathworks Inc., MA, USA). The maximum frequency of the power spectral density is 1600 Hz (Nyquist frequency) as a result of the sampling rate.

### Motion capture system

Motion capture during balance trials was conducted using six motion tracking cameras (Oqus, Exave AB, GBG, Sweden). The motion tracking cameras were used to capture the trajectory of a single reflective marker (diameter 18 mm) placed on the right shoulder of the subjects. Additionally, the trajectory of the center of pressure (COP) of subjects during the balance trials was monitored using a Bertec force plate (FP4060-07; Bertec Corporation, OH, USA). The motion camera system and force plate delivered synced data to a connected desktop computer, where Qualisys Track Manager (Exave AB, GBG, Sweden) is used to collect and process the acquired data. The sampling rate for both the force plate and motion capture system was 200 Hz.

### Experimental methodology

Before commencing the experiments, subject vibration perception thresholds were determined independently for the narrow and wide bandwidth mechanical noise by setting the vibration intensity to the maximal value, then ramping down the intensity until the subject reported a lack of sensation. This process was repeated until a reproducible threshold was achieved^21,24^. Vibration intensity for each noise was then set to 80% of the respective threshold^21^. Subjects were then briefed about the required standing postures prior to the balance trials and were allowed to decline further participation in the experiment at any point.

**Figure 2:**
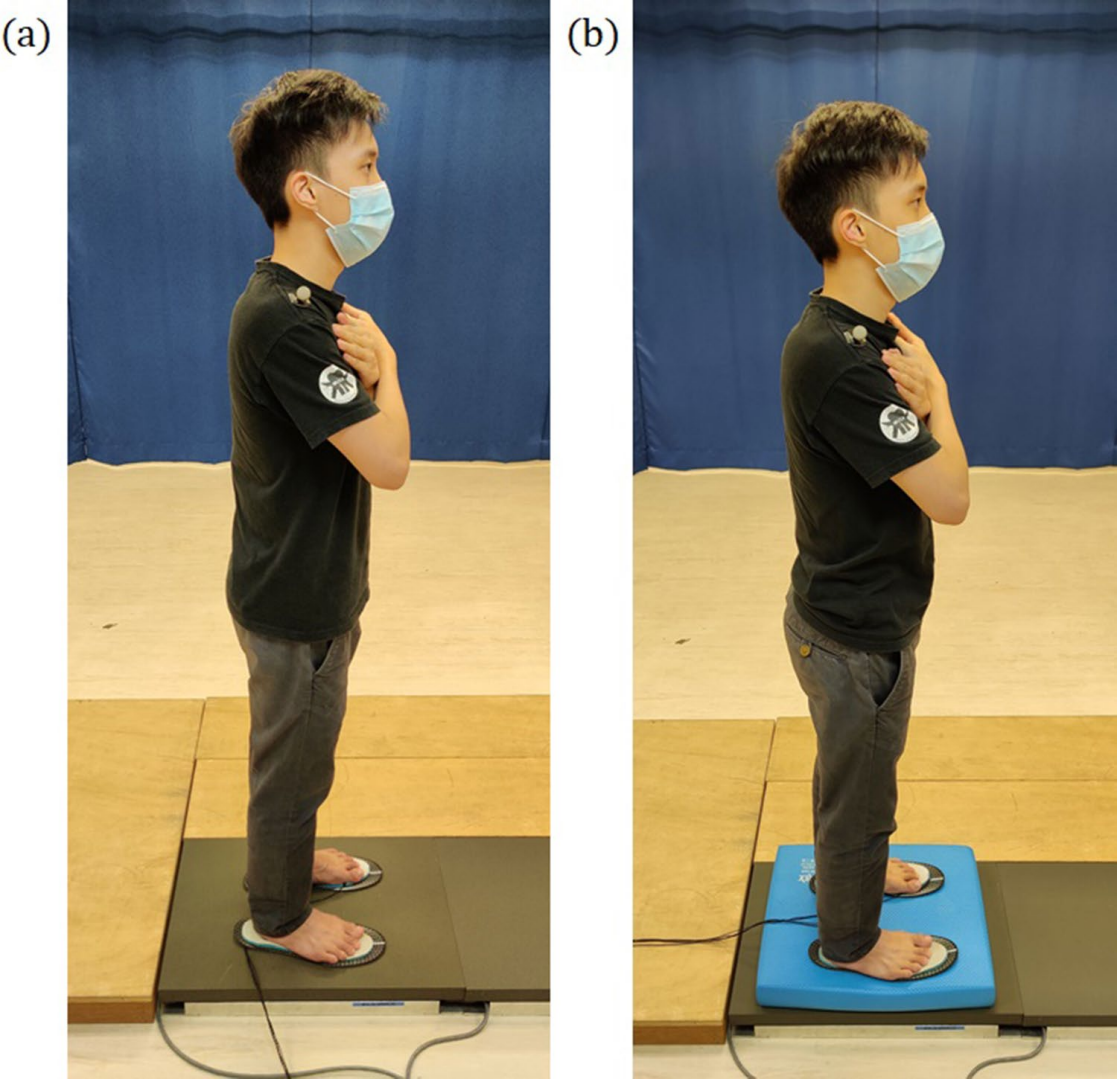
Subject posture during (**a**) firm surface and (**b**) compliant surface balance trials, with the reflective marker placed on the right shoulder.

During the balance trials, subjects were requested to stand on a firm surface with their eyes open, looking straight ahead and their feet positioned comfortably in a self-selected position. Additionally, subjects were required to keep their arms crossed over their chest with each hand touching the opposite shoulder. Subjects were also instructed to refrain from carrying out any unnecessary movement that could alter their posture during the trial. Unnecessary movement occurring during a trial led to the repetition of that trial. Subjects were allowed 5 s immediately before the start of each trial to assume the posture and verbally confirm their readiness for the start of the trial. This was done to ensure subjects were given ample time to acclimatize to the required standing posture. The same procedure was then repeated on the compliant surface. For the firm surface balance trial, subjects stood on the force plate. For the compliant surface balance trial, a foam exercise pad of $40\times 48\times 6$ cm (Balance Pad Elite; Airex AG, Sins, Switzerland) was placed on the force plate^32,33^. Subject posture during firm and compliant surface balance trials are shown in Fig. 2a,b respectively. Informed written consent was obtained for publication of the reference postures in Fig. 2.

A total of 15 data sets were acquired from each subject for the firm surface balance trials, and similarly for the compliant surface balance trials. Each data set was logged for 35 s. For each surface, subjects were tested under three different conditions: (1) wide bandwidth mechanical noise, (2) narrow bandwidth mechanical noise, and (3) sham stimulation (no vibration). Sham stimulation (no vibration) was provided as a control. Each type of noise was applied a total of five times in a single-blind randomized order to improve the reliability of the sway parameters measured^34^. Participants were given a four minute seated rest after the first nine balance trials, and again after the remaining six trials. This procedure was observed for both firm and compliant surface balance trials. All methods were carried out in accordance with relevant ethical guidelines and regulations.

### Data processing and analysis

The first 5 s of the 35-s recorded data, which allowed the system to settle down, was not used for analysis. The settling time of the measurement system was caused by the switching of the noise settings between control and different noise types. Removing the first 5 s allowed switch debouncing to be eliminated as a potential error source. The remaining data was then treated with a moving average filter of 15 frames to reduce the effects of extrinsic or intrinsic noise.

The processed data was then used to calculate the sway parameters of the subjects during the balance trials. The sway parameters assessed included the sway amplitude in the mediolateral (ML) and anterioposterior (AP) directions, swept area, and mean sway speeds in the ML and AP directions of the subjects^35^.

ML and AP sway ranges were calculated via the amplitudes (ranges) of the subject sway along the frontal and sagittal planes respectively (Eqs .1, 2). Swept area was calculated using the area of the 95% prediction ellipse (PEA) (Eq. 3), defined as the ellipse containing 95% of the points in the subject’s stabilogram. Mean sway speeds in the ML and AP directions were calculated by dividing the total path length of the COP or marker along the frontal or sagittal planes respectively by the total trial time^36^ (Eqs. 4, 5). The formulae used in the sway parameters calculation are listed below for a stabilogram of time duration *t* containing a set of *n* data points $(x_i,y_i)$ with eigenvalues of $\lambda _1$ and $\lambda _2$ from the co-variance matrix of the data.

1 $$\begin{aligned} \text {ML range}&= \max _{1\le i\le n} x_i - \min _{1\le i\le n} x_i \end{aligned}$$

2 $$\begin{aligned} \text {AP range}&= \max _{1\le i\le n} y_i - \min _{1\le i\le n} y_i \end{aligned}$$

3 $$\begin{aligned} 95\%\, \text {PEA}&= 5.991\pi \sqrt\lambda _1\sqrt\lambda _2 \end{aligned}$$

4 $$\begin{aligned} \text {ML speed}&= \frac{\sum _{i=1}^{n} |x_i - x_{i-1}|}{t} \end{aligned}$$

5 $$\begin{aligned} \text {AP speed}&= \frac{\sum _{i=1}^{n} |y_i - y_{i-1}|}{t} \end{aligned}$$

Calculated sway parameter metrics were normalized for data comparison. ML and AP sway ranges and mean velocities calculated from COP data were normalized to subject height (in meters), with swept area normalized to subject height squared. Similarly, sway ranges and mean velocities calculated from marker data were normalized to the height of the reflective marker (in meters), with swept area normalized to marker height squared. Statistical comparison between trial types was carried out via paired two-sided Student’s t-tests, with data normality confirmed using the Kolmogorov–Smirnov test (p < 0.05) on each trial data set. All data analyses were done using a custom MATLAB program.

## Results and discussion

### Mechanical noise bandwidth

**Figure 3:**
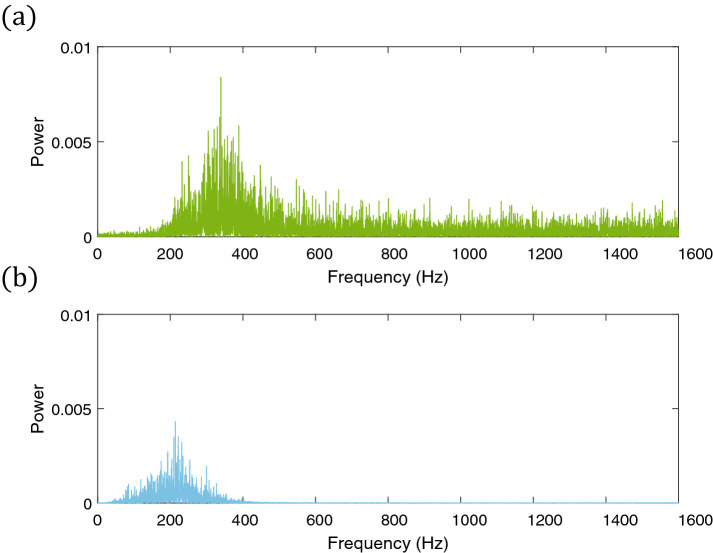
Power spectrum of the measured acceleration corresponding to the (**a**) wide and (**b**) narrow bandwidth mechanical noises.

Spectra of the measured acceleration corresponding to the narrow and wide bandwidth mechanical noises are shown in Fig. 3. From the figure, the effective bandwidth of the narrow bandwidth mechanical noise has a maximum frequency of approximately 400 Hz, while the wide bandwidth mechanical noise has frequencies extending up to the Nyquist frequency of 1600 Hz.

### Balance trial results

Mean (± SD) values for the COP and marker sway parameters are given in Tables 1 and 2 respectively, together with the p-values for the t-tests. For the firm surface, there was generally no statistically significant difference in the sway ranges, mean speeds, or swept area (COP and marker) for either noise type versus control and between the two noise types. The only exception is the sway speed in the ML direction (COP), where there was a significant difference between the results for the wide bandwidth noise and control – however, the mean value for the former was higher than the latter.

**Table 1:** COP sway parameters (mean ± SD) for firm and compliant surface balance trials with different mechanical noise bandwidths introduced.

Balance surface	Units	Parameter	Vibration bandwidth			P-values		
			Control (C)	Wide (W)	Narrow (N)	C versus W	C versus N	N versus W
Firm	mm	ML range	$7.1\pm 1.9$	$7.2\pm 1.8$	$7.0\pm 2.5$	0.854	0.938	0.792
		AP range	$12.4\pm 2.1$	$11.8\pm 2.8$	$11.6\pm 2.3$	0.428	0.382	0.823
	mm/s	ML speed	$2.3\pm 0.6$	$2.5\pm 0.8$	$2.4\pm 0.8$	**0.041**	0.339	0.074
		AP speed	$3.6\pm 0.4$	$3.7\pm 0.7$	$3.6\pm 0.6$	0.656	0.967	0.608
	mm$^2$	Swept area	$63.9\pm 27.5$	$62.7\pm 22.3$	$58.3\pm 25.1$	0.901	0.608	0.495
Compliant (foam)	mm	ML range	$17.0\pm 5.0$	$15.2\pm 4.2$	$16.2\pm 4.3$	**0.003**	0.193	0.095
		AP range	$23.2\pm 5.1$	$21.4\pm 5.4$	$22.8\pm 4.3$	0.201	0.610	0.184
	mm/s	ML speed	$5.1\pm 1.2$	$4.9\pm 1.0$	$5.0\pm 1.3$	**0.045**	0.099	0.384
		AP speed	$6.2\pm 1.0$	$5.8\pm 0.9$	$6.2\pm 1.2$	**0.009**	0.399	**0.036**
	mm$^2$	Swept area	$298.3\pm 129.7$	$248.1\pm 121.6$	$285.8\pm 124.1$	**0.046**	0.497	**0.009**

**Table 2:** Marker sway parameters (mean ± SD) for firm and compliant surface balance trials with different mechanical noise bandwidths introduced.

Balance surface	Units	Parameter	Vibration bandwidth			P-values		
			Control (C)	Wide (W)	Narrow (N)	C versus W	C versus N	N versus W
Firm	mm	ML range	$8.6\pm 2.0$	$8.9\pm 2.2$	$9.0\pm 3.5$	0.677	0.709	0.919
		AP range	$18.0\pm 3.0$	$16.7\pm 3.3$	$17.4\pm 3.6$	0.221	0.566	0.478
	mm/s	ML speed	$1.6\pm 0.4$	$1.7\pm 0.4$	$1.7\pm 0.6$	0.316	0.348	0.548
		AP speed	$2.9\pm 0.6$	$2.8\pm 0.6$	$2.8\pm 0.5$	0.416	0.399	0.963
	mm$^2$	Swept area	$148.8\pm 52.2$	$144.5\pm 50.3$	$140.9\pm 63.3$	0.838	0.734	0.828
Compliant (foam)	mm	ML range	$21.3\pm 6.3$	$19.1\pm 5.3$	$20.7\pm 5.8$	**0.017**	0.496	**0.034**
		AP range	$29.6\pm 7.0$	$28.5\pm 7.8$	$29.4\pm 5.9$	0.508	0.855	0.369
	mm/s	ML speed	$4.0\pm 1.0$	$3.7\pm 0.8$	$3.9\pm 1.0$	**0.043**	0.148	0.220
		AP speed	$5.2\pm 0.9$	$4.8\pm 1.0$	$5.0\pm 1.1$	**0.049**	0.297	**0.037**
	mm$^2$	Swept area	$598.6\pm 263.5$	$523.2\pm 261.1$	$607.8\pm 251.8$	0.156	0.828	**0.009**

For the compliant surface, there was no significant difference in all the sway parameters between the narrow bandwidth noise and control trials. However, there was significant difference in some of the parameters (COP: ML range, ML and AP speeds, and swept area; Marker: ML range, ML and AP speeds) for the wide bandwidth noise versus control, and in some of the parameters (COP: AP speed and swept area; Marker: ML range, AP speed, and swept area) for the wide bandwidth noise versus narrow bandwidth noise—in all cases, the mean value for the former was lower than the latter, indicating balance improved. And in all cases, the difference between the two mean values are all larger than the measurement error.

The measurement error for ML range, AP range, ML speed, AP speed and swept area are $\pm \,0.45$ mm, $\pm \,0.71$ mm, $\pm \,0.004$ mm/s, $\pm \,0.006$ mm/s, and $\pm \,2.89$ mm^2^, respectively. All the sway parameters were derived from COP data, which were in turn derived from force and moment measurements^37^. All errors were estimated using standard error propagation. The COP errors were estimated based on the errors in the force and moment measurements (0.02% of maximal rated value, which were obtained from the technical data sheet for the force plate). The sway parameter errors were estimated using a normal distribution of the COP errors, where the mean and standard deviation of the normal distribution were, respectively, the mean of the COP error range and a quarter of the range, based on the conservative assumption that the range contains 95% of the values (i.e. 2$\sigma$)^38^.

Our study showed reductions in sway parameters (approximately 4–17% across different parameters), of a consistent magnitude with those seen in the literature^15–17,21^, with the application of the wide bandwidth mechanical noise. The sway parameters are known to be correlated with balance ability—lower values better balance. In addition, a reduction in these sway parameters is correlated with a reduced fall risk among high fall risk groups^39,40^. Our findings thus point to a functionally improved balance when the wide bandwidth mechanical noise is applied.

### Model and proposed explanation

An important role of the somatosensory system in maintaining balance is to sense changes in pressure during balance via the mechanoreceptors in the feet. Consider the application of a pressure stimulus to a single mechanoreceptor in the skin. The pressure stimulus opens mechanically-gated Na+ channels in the membrane of the mechanoreceptor and initiates a depolarization of the membrane electric potential. However, to produce an action potential, the depolarization has to exceed a threshold potential value. This implies that the pressure stimulus has to exceed a pressure threshold to trigger the mechanoreceptor to produce an action potential (an electrical impulse). This action potential, which encodes the pressure stimulus, is transmitted as a nerve impulse to the brain for balance control. If the pressure stimulus is sub-threshold, no action potential is produced and therefore no information about the pressure stimulus is transmitted to the brain.

**Figure 4:**
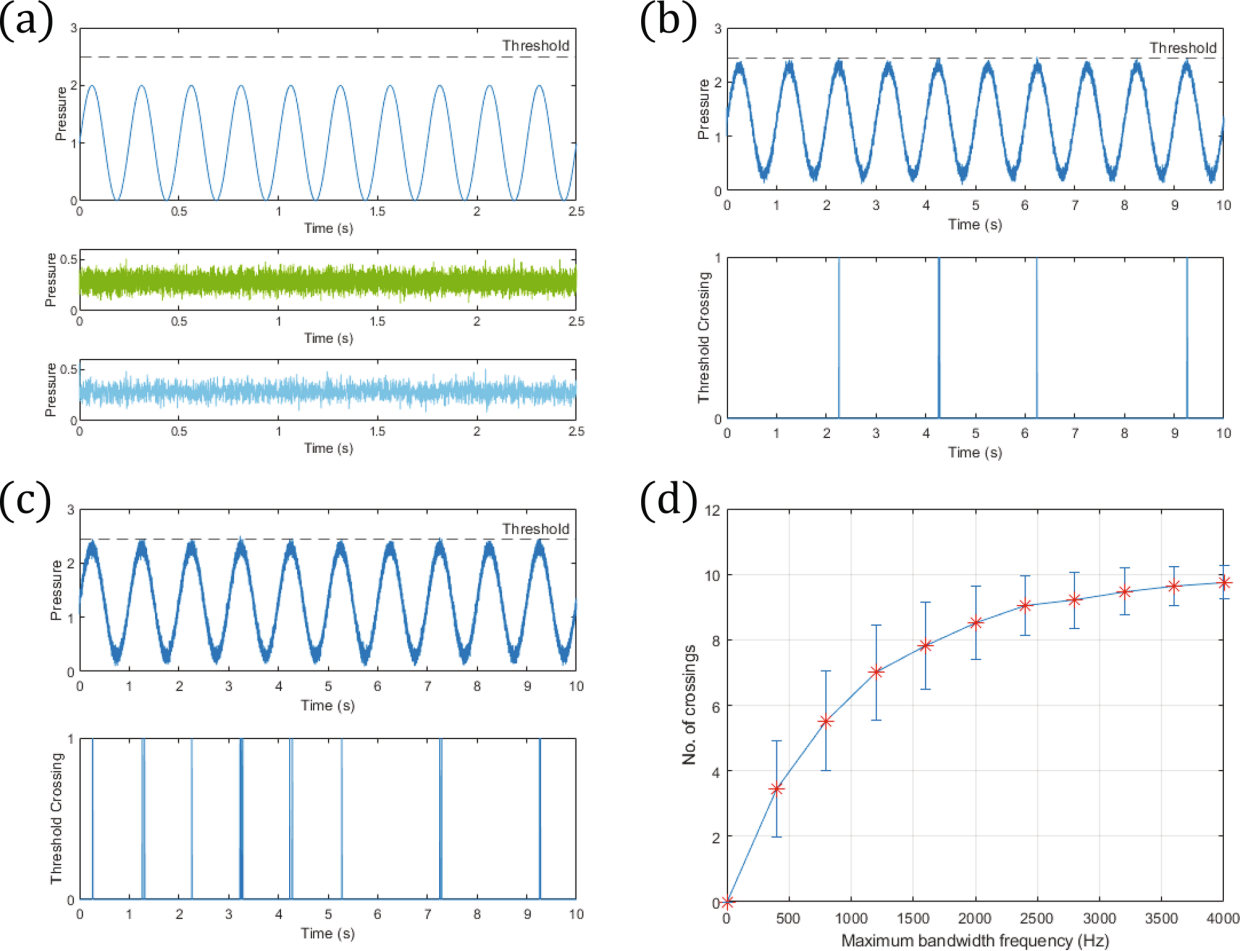
(**a**) Sub-threshold pressure stimulus (4 Hz sinusoid) and the chosen threshold (dotted line). A wide (maximum frequency 1600 Hz) and narrow (maximum frequency 400 Hz) bandwidth Gaussian noise is plotted below the sinusoid in green and blue, respectively. (**b**, **c**) The resultant pressure after the narrow and wide bandwidth Gaussian noise in (**a**) were added to the sub-threshold pressure, respectively. When the threshold is crossed by the resultant pressure, a spike of value 1 is plotted. (**d**) The number of crossings (over 10 cycles) versus the maximum bandwidth frequency of the noise. The plotted number of crossings for each maximum frequency is the mean of the number of crossings for 1000 realizations of the noise. The error bar is the corresponding standard deviation.

To help us understand the experimental results, we use a simple model for a sub-threshold pressure stimulus and the added mechanical noise to study the effect of varying the noise bandwidth on the resultant pressure stimulus. Since pressure changes in the foot are typically low frequency, we model the sub-threshold pressure stimulus as a 4 Hz positive sinusoid^41–43^, with values arbitrarily chosen to range between 0 and 2 (Fig. 4a). Gaussian noise, with maximum frequency ranging from 400 Hz to 4000 Hz, was used to model the experimental mechanical noise. To ensure that the resultant pressure is fully positive (as pressure cannot be negative), the Gaussian noise, with zero mean and variance $\sigma ^2$ (where $\sigma = 0.056$), was truncated to $\pm \,5\sigma$ and offset by $5\sigma$ before it was added to the sub-threshold pressure stimulus. The noises with maximum frequencies 400 Hz and 1600 Hz are plotted in Fig. 4a.

The resultant pressure stimulus (sub-threshold stimulus plus noise) for the two noise bandwidths (maximum frequency 400 Hz and 1600 Hz) is shown, respectively, in Fig. 4b,c. The addition of noise to the sub-threshold pressure enables the resultant pressure to cross the threshold occasionally, modelled here as a spike of value 1. In other words, the sub-threshold pressure is enhanced by the noise—this could be broadly defined as “stochastic resonance”^44^. Comparing the narrow bandwidth noise (maximum frequency 400 Hz) in Fig. 4b to the wide bandwidth noise (maximum frequency 1600 Hz) in Fig. 4c, it is clear that the frequency of crossing is low for the former but much higher for the latter. Moreover, increasing the maximum bandwidth frequency (from 400 to 4000 Hz) increases the frequency of threshold crossing, as shown in Fig. 4d. A higher frequency of threshold crossing by the resultant pressure stimulus leads to a higher frequency of action potential produced by the mechanoreceptor, and therefore a higher frequency of nerve impulse transmitted to the brain for balance control.

Our model suggests that, in both the firm and compliant surface experiments, although the addition of mechanical noise to a sub-threshold pressure stimulus results in some transmission of nerve impulses to the brain for both the wide and narrow bandwidth noise, the frequency of transmission is negligible for the latter but significantly higher for the former. For the compliant surface experiment, this would explain why the wide but not narrow bandwidth noise improved balance compared to control since the increase in frequency of nerve impulse transmission is significant only for the former. The same explanation applies to why balance is improved for the wide bandwidth noise compared to the narrow bandwidth noise.

For the firm surface experiment, the reason why similar balance improvements were not observed might be because the healthy young subjects already have near-optimal or optimal balance on this surface in the absence of noise. The frequency of nerve impulses transmitted to the brain is already sufficiently high in the absence of noise, and thus the brain has sufficient information to maintain optimal balance. Therefore, the increase in information sent to the brain with the application of the wide bandwidth mechanical noise does not result in any further improvement in balance. In contrast, a degraded somatosensation is mimicked^45,46^ on the compliant surface, which results in a comparatively lower frequency of nerve impulse transmission to the brain, and thus non-optimal balance for the same subjects. It is therefore possible to improve balance in this case with the addition of the wide bandwidth mechanical noise.

### Limitations

The main limitation of the current study was the small sample size. This study was intended as a first effort hypothesis-generating study. As such, we chose a similar sample size to other first-effort research in the field^16,17,19^. Nevertheless, to minimize the effects of the small sample size, we increased the reliability of the sampled results by increasing the number of intra-subject repetitions per test case to improve the intra-class correlation coefficient (ICC) of the data sample in accordance with recommended guidelines^34^. Future study will use a larger sample size to validate our present study and test our hypothesis. Additionally, due to our small sample size, we did not correct for multiple comparisons to avoid reducing the statistical power of our study and artificially inflate the probability of type II errors.

The aim of our study was to show that increasing the maximum frequency of the bandwidth of mechanical noise introduced would result in further improvements in balance. However, for a more controlled comparison, the two bandwidths should be similar but with different maximum frequency, unlike the two types of noise we used in this study, which differ in both width and maximum frequency.

## Conclusion

In conclusion, our experimental results for the compliant surface show that mechanical noise with different bandwidths have different effects on balance. In particular, the application of wide, but not narrow, bandwidth mechanical noise to the plantar side of the feet improved balance. This could be explained using a simple model, which suggests that adding the wide bandwidth noise to a sub-threshold pressure stimulus results in a significantly higher frequency of nerve impulse transmitted to the brain compared to adding the narrow bandwidth noise, which has negligible effect. However, we did not observe similar balance improvement on the firm surface when wide bandwidth noise was used. We surmise the reason for this is likely because the healthy young subjects can balance near-optimally or optimally on this surface by themselves.

Our results suggest that if a person’s standing balance is compromised (which can occur due to various factors such as age, injury, or disease), introducing a wider bandwidth mechanical noise, particularly with a higher maximum frequency, to the soles of the feet can improve the sensitivity of the feet to changes in pressure, resulting in improved balance. This is because a higher frequency of nerve impulse is transmitted to the brain. Aging increases the pressure threshold of a mechanoreceptor^47^ and thus a healthy elderly person is not able to balance optimally on a firm surface like a healthy young person. We hypothesize that repeating our experiment with healthy elderly subjects will show significant improvement in balance for both firm and compliant surfaces using the wide bandwidth mechanical noise.

As an extension of our study, future research should investigate the effects of different frequency ranges with a fixed bandwidth of the mechanical noise, allowing for the investigation of the influence of just maximum frequency on balance. Although studies have shown positive effects of mechanical noise on dynamic balance (like walking)^21^, future work should assess the potential of a wearable vibratory (high-maximum-frequency bandwidth) device that could be used long-term in dynamic conditions such as walking. Studies have also shown that the application of electrical noise can produce balance improvement lasting over several hours after the cessation of the electrical noise^33^. However, the possibility of residual effect has not yet been studied in the context of mechanical vibration.
